# Evaluation of sampling and sample preparation methodologies for determination of mercury concentrations and stable mercury isotopes in foliage samples

**DOI:** 10.1007/s10661-025-14318-6

**Published:** 2025-07-04

**Authors:** Saeed Waqar Ali, Dominik Božič, Sreekanth Vijayakumaran Nair, Igor Živković, Teodor-Daniel Andron, Stefan Marković, Marta Jagodic Hudobivnik, Milena Horvat, David Kocman

**Affiliations:** 1 Department of Environmental Sciences, Jožef Stefan Institute, 1000 Ljubljana, Slovenia; 2 Jožef Stefan International Postgraduate School, 1000 Ljubljana, Slovenia

**Keywords:** Measurement comparability, Mercury, Foliage sampling, Pre-treatment, Isotope analysis

## Abstract

Globally, foliar uptake of atmospheric mercury (Hg) is a key deposition pathway for terrestrial surfaces. However, diverse sampling and pre-treatment methods across studies can influence the comparability of foliar Hg concentrations and isotopic measurements, leading to biases that impact our understanding of Hg inter-compartmental exchanges at regional and global scale. In this study, we tested the effect of different sample drying and rinsing methods on foliage samples collected from three distinct locations on the tree crown of a European hornbeam (*Carpinus Betulus L.*). Samples were taken from Ljubljana, an urban area, and Idrija, a historically Hg-contaminated mining site, representing contrasting environments. Vertical crown stratification significantly influenced Hg concentrations, with inner canopy foliage in Ljubljana containing 46–50% higher Hg than outer and upper positions due to reduced solar exposure and enhanced throughfall deposition. Drying methods did not alter Hg concentrations (*p* > 0.05), validating cross-study comparability for low-Hg environments. Conversely, rinsing removed 24–60% of Hg in Idrija samples (*p* < 0.01), attributable to particulate-bound Hg (PBM) enriched in heavier isotopes (δ^20^^2^Hg: -1.9 ± 1.5‰ unrinsed vs. -3.2 ± 1.0‰ rinsed). We recommend that rinsing be applied when studying internal plant Hg cycling, but omitted when total foliage-associated Hg, including surface-bound fractions, is of interest. Future studies should investigate inter-species differences, seasonal variability in foliar Hg dynamics, and the effects of drying methods on Hg isotopic fractionation to improve methodological consistency and understanding of Hg cycling.

**Supplementary Information:**

The online version contains supplementary material available at 10.1007/s10661-025-14318-6.

## Introduction

Foliar Hg uptake is an important factor controlling seasonal atmospheric Hg concentrations and the associated Hg isotopic signatures (Fu et al., 2019; Jiskra et al., 2018; Pleijel et al., 2021; Zhou et al., 2021). Hg concentrations in foliage samples have been utilized for the estimation of vegetation Hg uptake fluxes (Wohlgemuth et al., 2020, 2022, 2023) and subsequently, for the evaluation of modelling outputs (Feinberg et al., 2022). The accuracy of this bottom-up approach is contingent on comparable estimations of Hg concentrations in foliage samples in various studies. This is particularly important since several approaches exist in the literature to quantify Hg concentrations in foliage samples. The diversity of these approaches stems primarily from the absence of generally accepted, validated, and standardized protocols for sample collection, preparation, and Hg quantification. This lack of standardization introduces significant uncertainties when comparing results from different foliar Hg assessment studies, thereby influencing the study of the role of vegetation in Hg intercompartmental exchanges. Consequently, it is crucial to evaluate the impact of these varied approaches to understand the uncertainties introduced for accurate estimation of Hg concentrations in foliage samples.

Foliage acts as an important sink for atmospheric Hg (Fay & Gustin, 2007; Wohlgemuth et al., 2020; Wang et al., 2020; Wohlgemuth et al., 2023; Yu et al., 2020) with studies showing a substantial portion of accumulated Hg being sequestered within internal tissues as Hg-sulfur nanoparticles within specific cellular compartments (Manceau et al., 2018; Sun et al., 2021). While the exact percentage may vary depending on plant species and environmental conditions, this internal sequestration can account for a major fraction of total foliar Hg. This sequestration is facilitated by oxidation processes with catalase activity playing a critical role (Tian et al., 2025). Following this, foliar Hg undergoes translocation through vascular bundles to different parts of the tree. It has been shown that this process accounts for approximately 67%, 80%, and 77% of Hg found in bole wood, branches, and bark, respectively (Liu et al., 2021). The declining concentrations of Hg from the top to the bottom of the bole and from older to newer tree rings also suggest that foliar Hg uptake plays a more significant role than root uptake (Yanai et al., 2020). However, species-specific differences in internal translocation processes, such as phloem-to-xylem transfer efficiency and canopy gas exchange duration, play a crucial role in determining tree-ring Hg content (Gačnik & Gustin, 2023; Liu et al., 2021; Yanai et al., 2020). Additionally, the mass-dependent fractionation (MDF) during translocation through vascular bundles is not fully understood and therefore remains a critical consideration when using tree rings as proxies for historical atmospheric Hg concentrations (Gačnik & Gustin, 2023; Liu et al., 2024; Liu et al., 2021; Peng et al., 2024; Xiao et al., 2025).

PBM and reactive gaseous Hg^2+^ deposited through wet and particle deposition are further complexed with carboxylate groups on leaf surfaces (Sommar et al., 2020; Sun et al., 2021). Although lower than the amount of Hg^0^ taken up into the leaf's internal tissue, the deposited Hg on the foliar surfaces may account for an important fraction of the total Hg concentration quantified in foliage samples (Stamenkovic & Gustin, 2009), particularly in regions with higher deposition of PBM (Zhang et al., 2019). Furthermore, different parts of individual trees are subjected to varying degrees of atmospheric Hg deposition (B. Wang et al., 2022). Foliar Hg uptake rates normalized to leaf area have been shown to be highest at the top of the tree crown, indicating differences in Hg uptake and deposition within the tree crown (Wohlgemuth et al., 2020). This variation in foliar Hg uptake is further reflected in the distinct isotopic signatures observed within different canopy layers. It has been shown that air within the canopy has significantly higher MDF values relative to the air above the canopy, due to the preferential uptake of lighter isotopes by vegetation (Wang et al., 2022). Similarly, the inner part of the canopy has been shown to exhibit significant negative odd mass-independent fractionation (odd MIF) values compared to the upper part and above the canopy (Wang et al., 2022). Significant Δ^199^Hg observed in the air within the inner part of the tree canopy compared to the upper canopy and above-canopy air can be attributed to contributions from soil and litterfall emissions near the forest floor, which release Hg⁰ with distinct negative Δ^199^Hg signatures due to microbial and abiotic reduction processes. Additionally, reduced atmospheric mixing within the lower canopy traps isotopically distinct Hg⁰ from these sources, while bi-directional exchange processes, such as reduced re-emission relative to uptake, further contribute to retaining more negative Δ^199^Hg values in this zone (Wang et al., 2022). The quantification of foliar Hg concentrations and the associated Hg isotopic signatures could therefore, be influenced by the methodology employed for foliage sample collection during field campaigns leading to inaccurate interpretations of the related mechanisms.

Sample drying is a critical procedure for achieving a constant foliage sample weight and several methods have been used to dry foliage samples prior to Hg concentration quantification. The most common methods for sample drying include oven drying (Du et al., 2019; Hellings et al., 2013; Ma et al., 2022), drying at room temperature (Wang et al., 2016; Yusupov et al., 2022; Zhou et al., 2013), and freeze-drying (Ericksen et al., 2003; Gong et al., 2014; Obrist et al., 2012) followed by homogenization into fine powdered form. While oven drying at higher temperatures potentially increases Hg volatilization, air-drying foliage samples at room temperature increases the chances of contamination from ambient indoor air (Anawar et al., 2011; Yang et al., 2017). Nevertheless, the comparability of results from foliage samples subjected to different drying procedures has not been evaluated. This is particularly important in the context of how such procedures have the potential to influence the foliar Hg content and isotopic signatures.

The evaluation of sample rinsing effect on foliar Hg quantification and associated isotopic signatures addresses the potential influence of foliar surface-bound Hg on overall measurements. This is crucial for distinguishing between internally assimilated Hg and externally deposited PBM, especially in areas with high particulate deposition. Previously, the effect of sample rinsing on Hg dynamics in foliage was evaluated through leaf washing experiments under controlled conditions, in the context of distinguishing between dry deposition and foliar leaching processes. It was shown that most Hg, along with other elements like lead (Pb), cadmium (Cd), lanthanum (La), and cerium (Ce), is removed rapidly from foliage, typically within 30 min (Rea et al., 2000). While studies on the effect of sample rinsing on foliar Hg concentrations are scarce, the influence of sample rinsing on foliar Hg isotopic signatures remains unexplored. Understanding this influence is essential, as Hg isotopic signatures can provide insights into the sources and cycling of Hg in forest ecosystems. Despite advances in understanding foliar Hg dynamics, inconsistencies in methodological approaches remain a critical gap. This study seeks to address this gap by evaluating how different sampling and pre-treatment methods influence both Hg concentrations and isotopic signatures, particularly in regions with contrasting Hg sources. By systematically evaluating different drying methods and rinsing procedures, the current study provides a comprehensive framework for identification of potential discrepancies introduced by sample preparation procedures, which is critical for ensuring the accuracy and comparability of Hg measurements across multiple studies.

## Methodology

### Study area and sampling

Two sites in Slovenia were selected to represent distinct Hg presence in the environment. The first site is Idrija, a town with more than 500 years of Hg mining history, where Hg concentrations have been found to be elevated in the air (Kocman et al., 2011; Kotnik et al., 2005) and soil, even though mining activities ceased in 1995 (Floreani et al., 2023; Kocman et al., 2004; Tomiyasu et al., 2017). The area surrounding the sampling site sits on top of the sedimentary carbonate rocks bearing Hg deposits. Historical mining activities have released Hg into the surrounding soil via wet and dry deposition for centuries and continue to do so from mine tailings. Mine tailings at former mining sites, such as Idrija, continue to act as persistent sources of atmospheric Hg emissions through volatilization and erosion processes. These tailings, often enriched in Hg due to historical mining activities, are exposed to climatic conditions such as solar radiation and precipitation, which enhance Hg volatilization and dispersion into surrounding environments (Kocman et al., 2011). Studies have shown that abandoned cinnabar tailings can release significant amounts of Hg⁰ into the atmosphere and contribute to elevated Hg concentrations in nearby soils and sediments (Barago et al., 2023; Zhao et al., 2023). The dispersion of these tailings through wind and water erosion further exacerbates contamination in adjacent terrestrial and aquatic systems (Kocman et al., 2004; Tomiyasu et al., 2017). As a result, the surrounding environment is extensively contaminated in Idrija. Sampling in Idrija was conducted in a forest stand near the former smelter area (Topilnica), which historically served as a major site for Hg ore processing. Although mining and smelting activities ceased in 1995, the area remains extensively contaminated due to centuries of cinnabar processing and atmospheric deposition of Hg-rich particles. Recent efforts have focused on preserving and restoring parts of the smelter as part of Idrija's UNESCO World Heritage designation, but environmental contamination persists in surrounding soils and vegetation (Gosar et al., 2016). In contrast, the city of Ljubljana is an urban setting with diffuse Hg sources, including industry, traffic, and the use of Hg-containing products (Gosar et al., 2016).

**Fig. 1:**
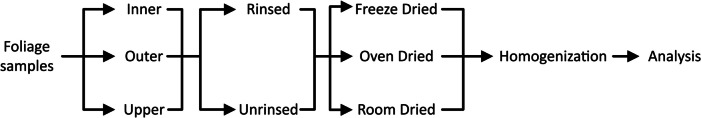
Schematic diagram of foliage sampling and sample pre-treatment methods tested. Tests comprised of 18 unique combinations of crown position (inner, outer and upper), sample drying (room, oven and freeze), and rinsing (rinsed and unrinsed)

Foliage samples collected from different locations on the tree crown underwent selected pre-treatment procedures before the analysis, as shown in Fig. 1. Mature, healthy European hornbeam (*Carpinus betulus* L*.*) trees (n = 3) of similar size and age were selected from each site. Foliage samples were collected from the two sites during mid-June 2021, with sampling conducted in Ljubljana on June 14th and in Idrija on June 21st. This narrow timeframe minimized seasonal variability in foliar Hg concentrations, which are known to increase over the growing season in deciduous trees (Laacouri et al., 2013). *Carpinus betulus* L., a native European hornbeam species, was selected due to its ecological relevance and widespread distribution in temperate forests. The leaves of *Carpinus betulus* L. are characterized by a dense tissue structure with both palisade and spongy mesophyll layers, which contribute to efficient photosynthesis and particulate trapping. Studies have shown that species with waxy surfaces or trichomes, such as *Carpinus betulus* L. exhibit substantial capacity for intercepting atmospheric particulates, including PBM (He et al., 2020; Sæbø et al., 2012; Steinparzer et al., 2022).

Individual tree crowns were divided into three positions (inner, outer, and upper) as shown in Fig S1 (Online Resource 1). Two sets of fresh foliage samples (~ 200 g each) were collected from each position on the tree crown and sealed in separate air-tight bags. Foliage samples were composite samples formed by collecting material from multiple branches within each designated section. The inner part was defined as those that were not directly exposed to the open air and are closer to the tree trunk. The outer part included those that were on the exterior part of the tree crown, whereas the upper part consisted of the top part of the crown that was directly exposed to open air. Pre-cleaned pruners were used to collect samples from different parts of the crown and a ladder was used to access the upper part of the tree crown.

### Sample preparation

Since 2 sets of foliage samples were collected for each position on the tree crown of individual trees, one set of foliage samples was thoroughly rinsed with ultrapure Type I water (resistivity 18.2 MΩ·cm at 25 °C, Milli-Q system), whereas the other set of the same foliage samples was kept unrinsed. The rinsing procedure involved placing the samples in a plastic bowl lined with a mesh. The foliage samples were submerged three times in fresh Milli-Q water for 1 min, ensuring complete wetting during each submersion. On the fourth rinse, Milli-Q water was allowed to run over the samples for approximately one minute before draining them thoroughly. On average, 2 L of Milli-Q water were used for 200 g fresh foliage samples. This approach was designed to remove loosely adhered PBM effectively while minimizing potential leaching of Hg from internal leaf tissues.

The leaves of *Carpinus betulus* are known to have waxy surfaces and hairs (trichomes) on their lower surface, which enhance their capacity to trap atmospheric particulates, including PBM (He et al., 2020; Sæbø et al., 2012). These structural features may also influence how particles are retained and subsequently removed during rinsing. The thorough rinsing protocol used in this study aimed to account for these characteristics by ensuring that surface-bound particles were adequately removed without damaging the leaf structure or altering internal Hg content.

All samples, including both rinsed and unrinsed, were randomly divided into three equal parts. One part, comprising both rinsed and unrinsed samples, was then placed in sample containers and freeze-dried at -60 °C. The second set of foliage samples was dried in the dryer at 60 °C for 24 h, while the third set of foliage samples was dried in a clean room for several weeks until a constant weight was achieved. Drying was performed separately for the rinsed and unrinsed samples and for each site to avoid possible cross-contamination. The samples were then ground into fine particles in their respective containers using a mortar and pestle. The sample containers were placed in resealable Ziploc bags at room temperature for further analysis. In total, tests were performed on 18 unique combinations of crown positions (inner, outer, and upper), sample rinsing (rinsed and unrinsed), and sample drying methods (room dry, oven dry, and freeze dry) from each sampled tree.

For residual moisture determination, representative 1 g subsamples from each foliage treatment (rinsed and unrinsed, processed by room drying, freeze drying, or oven drying) were weighed into glass vials that had been pre-dried in an oven at 105 °C for 30 min to remove any residual moisture. The prepared vials were then sealed and placed in a vacuum desiccator containing magnesium perchlorate as a desiccant, thereby ensuring a controlled, low-humidity environment for drying. Samples were initially dried in the desiccator for 120 h, after which they were weighed, returned for an additional 48 h of drying, and weighed again. When the difference in mass between successive measurements was less than 5 mg, the sample was considered to have reached constant weight. Moisture content was calculated using the dry factor approach (Yang et al., 2017), whereby percentage moisture content is derived from the weight loss relative to the final, constant dry weight.

### Sample analysis

#### Hg concentrations and isotope analysis

Hg concentrations in foliage samples were analyzed following a previously reported method (Božič et al., 2022). Briefly, samples were digested using a microwave digestion system (UltraWave, Milestone, Italy). About 0.3–0.4 g of each sample was weighed in pre-cleaned polytetrafluoroethylene (PTFE) tubes. Then, 3 mL HNO_3_ (65% Suprapur), 0.02 mL HCl (37% Suprapur) and 0.02 mL HF (40% Suprapur) were added. The samples underwent closed-vessel microwave digestion for 90 min at a maximum power of 1500 W and a pressure of 100 bar. The solution was quantitatively transferred into 10-mL polyethylene graduated tubes and diluted with Milli-Q to 10 mL. Prior to the measurements, two sets of dilutions were prepared using 0.5 mL and 1 mL of the digested samples, and the final volume was adjusted to 10 mL using 5% HNO_3_.

Freeze-dried foliage samples from both sites were prepared and digested specifically for Hg isotope analysis. Foliage samples with concentrations above 40 ng g^-1^ were digested using a microwave digestion system (UltraWave, Milestone, Italy) to obtain approximately 1 ng g^-1^ of Hg in the solution, following the protocol used for Hg analysis. Foliage samples with lower Hg concentrations required a preconcentration step and were therefore prepared using a novel preconcentration method (Ali et al., 2024). Briefly, up to 2 g of foliage samples were carefully weighed and placed in pre-cleaned PTFE digestion vessels. The samples were pre-digested through the sequential addition of 10 mL of HNO_3_ (65% Suprapur), 1 mL of HCl (37% Suprapur), and 1 mL of H_2_O_2_ (37% Suprapur). The digestion process was carried out using a microwave digestion system, ETHOS 1 (Milestone Inc., Shelton, CT, USA). After digestion, the vessels were allowed to cool before being rinsed with Milli-Q and dried for subsequent use in sample digestion. Microwave-digested foliage samples and certified reference materials (NIST SRM 1575a, NIST SRM 1547) were transferred to an impinger in which Hg was reduced with SnCl_2_, purged with nitrogen gas, and Hg was captured in a 2.25 mL concentrated solution of inverse aqua regia (3:1 v/v). Following the dilution to 15 mL with Milli-Q water, acid concentration in the solution was optimal for Hg stable isotope analysis.

Hg concentrations in the pre-concentrated freeze-dried samples were determined using a cold vapor atomic absorption spectrometer (CV-AAS) (Automatic Hg Analyzer Model Hg-201, Sanso Seisakusho Co., LTD). The instrument was calibrated using NIST SRM 3133 using a single-point calibration (1 mL of 1 ng/mL working standard solution). The instrument was warmed up for at least 60 min prior to analysis, and the condensation tube was kept chilled with ice packs to ensure vapor condensation. Prior to each batch of sample analysis, blanks were checked regularly for each digestion vessel. Sample blanks corresponded to 5% ± 1.8% (1SD, *n* = 12) of Hg in the trapping solution. For analysis, sample aliquots were pipetted into the reaction chamber, reduced with 1 mL of 10% SnCl_2_ (v/v), purged with air, and detected by AAS. Blanks (calibration and sample) were measured prior to each sample measurement along with calibration standards and samples.

Following the required dilutions, Hg stable isotope analyses were performed using a Nu Plasma II (Nu Instruments Ltd., Ametek, UK) Multi-Collector Inductively Coupled Plasma Mass Spectrometer (MC-ICP-MS) following a previously reported method (Božič et al., 2022). A SnCl_2_ solution (3% w/v in 10% v/v HCl) was utilized to reduce Hg, which was combined with the sample using a T-piece. The resulting mixture was directed into a Tekran frosted glass rod phase separator (Part Number: 35–26300-00) achieving a Hg reduction recovery rate of up to 99.6%. Elemental Hg vapor was carried to the instrument by an argon sweep gas flow. The intensity readings at m/z 202 ranged from 0.5 to 1.2 V, corresponding to a Hg solution concentration of 1 ng/mL, with an uptake rate of 1 mL/min. For each cycle, 60 measurements were conducted with an integration time of 10 s. The Hg concentrations in samples and bracketing standards (NIST SRM 3133) were matched within 10% uncertainty, minimizing concentration-dependent mass bias effects.

To align our findings with existing literature, Hg isotope ratio measurements were performed utilizing the standard–sample–standard bracketing technique as detailed by (Blum & Johnson, 2017). Outlier rejection was applied to individual measurements within the cycle for values surpassing three relative standard deviations (RSD). The results are expressed using δ and ∆ notations (Blum et al., 2014). The δ values of the Hg isotopes are expressed in parts per thousand (‰) and represent the ratio of the Hg isotopes relative to NIST SRM 3133, as shown in Eq. 1. While all Hg isotopes except ^196^Hg were measured in this study, we focused on δ^20^^2^Hg and Δ^199^Hg as they are the most relevant parameters in the literature for evaluating the effects of sample pretreatment methods (drying and rinsing) on Hg isotopic signatures.

1 $${\updelta }^{xxx}Hg=\left(\frac{\frac{{xxx}_{{Hg}_{sample}}}{{198}_{{Hg}_{sample}} }}{\frac{{xxx}_{{Hg}_{SRM 3133}}}{{198}_{{Hg}_{SRM 3133}}}} -1\right)\times 1000$$

The Δ values were derived from the measured δ^xxx^Hg values using the formula: Δ^xxx^Hg ≈ δ^xxx^Hg - δ^202^Hg × f, where xxx represents the mass number of the isotope. The correction factors used were 0.2520 for ^199^Hg, 0.5024 for ^200^Hg, 0.7520 for ^201^Hg, and 1.4930 for ^204^Hg. Measurement uncertainty is reported as the long-term reproducibility of the measurements conducted with NIST SRM 8610 (UM-Almadén), with all uncertainties expressed at a coverage factor of k = 2.

### LA-ICP-MS analysis

Selected foliage samples from Idrija were prepared for bioimaging to perform spatial analysis for the effect of sample rinsing on Hg distribution in foliage samples. The spatial analysis of foliage samples from Ljubljana was constrained by low Hg concentrations. Unrinsed foliage samples were split into equal halves using a precleaned scalpel. One half of the sample was rinsed using the sample rinsing protocol used for Hg analysis, while the other half was kept unrinsed. Both sections of the sample were subsequently fixed on glass slides using double-sided scotch tape to achieve a flat surface and dried for 30 min before analysis by LA-ICP-MS following the protocol reported elsewhere (Marković et al., 2022).

For the mapping of Hg in the sample, an Analyte G2 193 ArF excimer laser ablation system, integrated with a HelEx II low-dispersion ablation cell (Teledyne Photon Machines Inc., Bozeman, MT, USA), was coupled to an Agilent 8800 QQQ-ICP-MS (Agilent Technologies Inc., Tokyo, Japan) through the Aerosol Rapid Introduction System (ARIS) provided by Teledyne Photon Machines. The HDIP LA imaging software (acquired from Teledyne Photon Machines) facilitated the generation of an elemental distribution map.

### QA/QC procedures

Quality control of the analytical methods was achieved by digesting certified reference materials NIST SRM 1575a (Pine Needle), NIST SRM 1547 (Peach Leaves), and NIST SRM 1515 (Apple Leaves) in each batch of sample digestion for Hg analysis, with average recoveries of 97% ± 8.5% (1SD, *n* = 18), 97% ± 18% (1SD, *n* = 22), and 102% ± 10% (1SD, *n* = 24), respectively. Procedural blanks were analyzed for each digestion batch. The limit of detection (LOD) for QQQ-ICP-MS, established at 0.001 ng g^-^^1^, was calculated by taking three times the standard deviation of the procedural blanks and dividing it by the slope of the calibration curve. The limit of quantification (LOQ) of the instrument was 0.003 ng g^-1^, which was calculated as 3.33 × LOD.

The LOD for CV-AAS was established at 0.03 ng, derived from three times the standard deviation of the procedural blank (n = 12). Similarly, the LOQ was determined to be 0.1 ng, calculated as 3.33 times the LOD. Following each digestion batch, the vessels were thoroughly rinsed with Milli-Q, and a clean-up procedure was conducted. This involved adding 10 mL of Milli-Q and 10 mL of HNO_3_ to each digestion vessel, followed by microwave digestion with a clean-up program that included a 15-min ramp-up to 200 °C. The impingers employed for Hg pre-concentration were thoroughly rinsed with Milli-Q between each pre-concentration run.

The LA-ICP-MS system was calibrated using NIST SRM 612 (Glass) to achieve equal operating parameters and sensitivity. Multiple parameters were tested for elemental mapping using LA-ICP-MS; however, the higher resolutions tested (spot size 2–10 µm) yielded low sensitivity and extended analysis times by manifold. For practical reasons, the spot sizes used for mapping were 35 µm squares, unless otherwise noted. ARIS enabled rapid analysis while ensuring good signal retention. A helium carrier gas was utilized at a flow rate of 0.7 L min^-1^ to transport the sample aerosol into the ICP, while argon make-up gas was supplied at 0.95 L min^-1^ through the ARIS mixing bulb. The washout time for a single laser pulse with these measurement parameters was approximately 60 ms. Given the extensive area of the foliage, measurement parameters for LA-ICP-MS imaging were optimized to ensure high resolution while balancing analysis time and sensitivity. A spatial resolution of 35 μm × 35 μm per pixel was achieved by configuring the laser beam to a square spot of 35 μm × 35 μm and adjusting the scanning speed to 350 μm s^-1^ with parallel line scanning. Due to the variable thickness of the foliage sample, the laser fluence was set at 3.5 J cm^-2^ with a firing rate of 100 Hz to optimize sample ablation. The dwell time per cycle was established at 100 ms; however, some regions within the veins were not fully ablated due to their thickness and limitations in laser focus.

Similarly, CRMs (NIST SRM 1575a (Pine Needle), NIST SRM 1547 (Peach Leaves)) were digested along with freeze-dried foliage samples that required Hg preconcentration for isotope analysis with recoveries of 95% ± 4.7% (1SD, *n* = 14) and 96% ± 5.6% (1SD, *n* = 2), respectively. NIST SRM 1575a (Pine Needle) had δ^202^Hg value of -1.2 ± 0.10‰, Δ^199^Hg -0.32 ± 0.04‰, Δ^200^Hg 0.02 ± 0.02‰, Δ^20^^1^Hg -0.35 ± 0.04‰, and Δ^20^^4^Hg -0.01 ± 0.04‰ (1SD, *n* = 14) (Ali et al., 2024), which aligns well with published literature values (-1.3 ± 0.14‰, 1SD, *n* = 5)) (Blum & Johnson, 2017; Enrico et al., 2021; Huang et al., 2019; Kurz et al., 2019; Zheng et al., 2016). Statistical test (t-test) showed no significant difference (*p* > 0.05) between these results and those reported in the literature. For NIST SRM 1547 (Peach Leaves), the reported values were as follows: δ^202^Hg -1.7 ± 0.02‰ (1SD, *n* = 2), Δ^199^Hg -0.13 ± 0.01‰ (1SD, *n* = 2), and Δ^200^Hg 0.01 ± 0.01‰, Δ^20^^1^Hg -0.17 ± 0.01‰, and Δ^20^^4^Hg -0.01 ± 0.07‰ (1SD, *n* = 2) (Ali et al., 2024). For NIST SRM 8610 (UM-Almadén), the reported values were δ^2^^0^^2^Hg -0.52 ± 0.08‰, Δ^199^Hg 0.00 ± 0.01‰, Δ^2^^00^Hg 0.01 ± 0.01‰, Δ^2^^0^^1^Hg -0.04 ± 0.02‰, and Δ^20^^4^Hg 0.01 ± 0.05‰ (1SD, *n* = 10) whereas, the average long-term values for NIST 3133 relative to itself were as follows: δ^2^^0^^2^Hg 0.03 ± 0.08‰, Δ^1^^99^Hg -0.02 ± 0.03‰, Δ^2^^00^Hg 0.00 ± 0.02‰, Δ^2^^0^^1^Hg 0.00 ± 0.03‰, and Δ^20^^4^Hg 0.04 ± 0.06‰ (1SD, n = 10)(Ali et al., 2024).Statistical analysis and visualisation.

A paired t-test was conducted to assess statistically significant differences in Hg concentrations between rinsed and unrinsed foliage samples collected from three distinct positions on the tree crown and dried using three different methods. Similarly, paired t-tests were used to evaluate statistically significant differences in residual moisture content for the same samples subjected to three different drying methods (room-drying, oven-drying, and freeze-drying). Visualizations were created using Systat SigmaPlot Version 14 and Tableau Desktop version 2023.2.

## Results and discussions

Across the 18 sample pre-treatment tests conducted, the average Hg concentration in the foliage samples from Ljubljana was 7.7 ± 4.1 ng g^-^^1^ (1SD, *n* = 48), while the average Hg concentration in the foliage samples from Idrija was 168 ± 149 ng g^-^^1^ (1SD, *n* = 48). The results of foliar position effects on Hg concentrations and sample pre-treatment analyses are presented separately for each site in the subsequent sections.

### Vertical distribution within tree crown in Ljubljana

In Ljubljana, significantly higher Hg concentrations were observed in the inner foliage samples than in the outer and upper samples in Ljubljana, as demonstrated by the in-canopy variation in foliar Hg concentrations (*p* < 0.05). Mean Hg concentration of the inner foliage samples across the different sample drying and rinsing tests were 12 ± 0.94 ng g^-^^1^ (1SD, *n* = 18) whereas concentrations for the outer and upper crown foliage samples were 8.7 ± 1.7 ng g^-^^1^ (1SD, *n* = 18) and 8.5 ± 1.2 ng g^-^^1^ (1SD, *n* = 18) respectively. The forest floor at Ljubljana site exhibited sparse vegetation, with the majority of the area being characterized by exposed soil. Studies indicate that soil Hg concentrations in Ljubljana are elevated, with mean values ranging from 250 ng g^-^^1^ to 410 ng g^-^^1^ (Gosar et al., 2016; Rodrigues et al., 2006). Soil contamination in Ljubljana is largely attributed to emissions resulting from traffic, industry, and households, particularly the utilization of fossil fuels for the generation of heat and power. Therefore, Hg re-emitted from contaminated soil surfaces can serve as a secondary atmospheric source for urban forests. This process may contribute to the higher Hg concentrations observed in inner foliage samples within Ljubljana. Furthermore, elevated Hg concentrations in the inner foliage samples may be attributed to factors such as reduced exposure to solar radiation within the tree crown, potentially resulting in decreased foliar Hg re-emissions (Obrist et al., 2018; Wang et al., 2022). The inner foliage samples are likely to receive higher throughfall Hg and are shielded from the washout of surface Hg by precipitation (Obrist et al., 2018; Wang et al., 2022). This interplay between reduced solar exposure and increased throughfall Hg suggests a complex relationship where inner foliage not only accumulates more Hg but also experiences altered biogeochemical cycles compared to outer and upper foliage. The vertical distribution of Hg concentrations in foliage samples was evaluated for the influenced of sample rinsing and drying methods and the results are presented in the following sections.

The MDF values for both sets of rinsed and unrinsed foliage samples from Ljubljana exhibited variability depending on their position within the tree crown (Fig. 3a), which may be attributed to natural variation in foliar Hg uptake and atmospheric exposure (Song et al., 2022; Wang et al., 2022). Forest canopies display a vertical gradient in MDF values, with positive values observed for air within the canopy compared with those above the canopy (Wang et al., 2022) owing to the preferential uptake of lighter Hg^0^ isotopes by foliage (Yuan et al., 2019). This is in line with our finding where foliage samples from Ljubljana demonstrated a broader range of negative MDF values relative to the MIF signatures which typically exhibit a more constrained range. The broader variation in MDF compared to MIF observed in our samples aligns with established isotopic behaviour in environmental matrices, where MDF occurs during diverse kinetic and equilibrium processes, while MIF is primarily associated with photochemical transformations of Hg^2^⁺ (Bergquist & Blum, 2009; Blum et al., 2014). The consistently negative MDF values observed in foliage samples reflect the preferential uptake of lighter Hg isotopes during stomatal assimilation of gaseous Hg⁰, while the minimal MIF signatures suggest that foliar Hg uptake is dominated by gaseous Hg⁰ rather than particulate or oxidized forms of Hg (Wang et al., 2022; Yuan et al., 2019).

On the contrary, negative MIF signatures in foliage indicate atmospheric deposition or origins associated with specific industrial emissions, whereas positive MIF signatures are often indicative of photochemical reactions occurring in the atmosphere or on surfaces, such as foliage (Sonke, 2011). In general, foliar Hg re-emission is linked to negative shifts in foliar MIF values relative to the atmosphere during in situ observations (Demers et al., 2013; Enrico et al., 2016; Olson et al., 2019) and controlled experiments (Yuan et al., 2019). Moreover, the re-emitted Hg^0^ from foliage exhibits positive odd-MIF signatures resulting from the reduction of Hg complexes that contain reduced sulfur functional groups (Demers et al., 2013). The odd-MIF (^199^Hg) in foliage samples from Ljubljana averaged -0.13 ± 0.04‰ (1SD, *n* = 9), which is in line with previous observations (Enrico et al., 2016; Yuan et al., 2019). The relatively small magnitude of Δ^199^Hg supports the conclusion that foliar Hg primarily originates from atmospheric Hg⁰, as photochemical processes associated with oxidized Hg species (Hg^2^⁺) typically produce larger positive MIF signatures (Blum et al., 2014; Wang et al., 2022). However, no variations were observed in the MIF signatures within the tree crown.

### Effect of sample drying

**Fig. 2:**
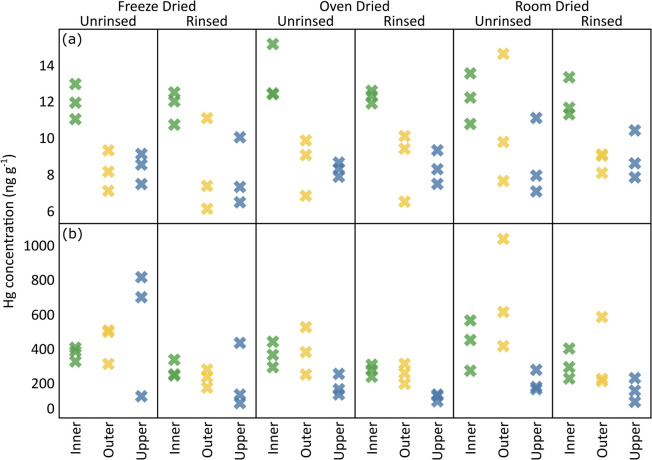
Foliar Hg concentrations (ng g^-1^) under varying drying conditions (freeze-dried, oven-dried, and room-dried) for rinsed and unrinsed foliage samples from (**a**) Ljubljana and (**b**) Idrija

Figure 2(3) shows vertical foliar Hg concentrations (ng g^-1^) under varying drying conditions (freeze-dried, oven-dried, and room-dried) for rinsed and unrinsed foliage samples. These drying methods were selected to evaluate their influence on Hg content in foliage samples collected from distinct positions on the tree crown, an aspect, which is critical for accurate environmental monitoring. Hg concentrations in Ljubljana samples demonstrated distinct patterns when comparing the different drying methods for each foliage position and sample pre-treatment category. For inner foliage samples in the rinsed category, Hg concentrations were identical between freeze-dried (12 ± 0.91 ng g^-1^, 1SD, *n* = 3) and oven-dried samples (12 ± 0.35 ng g^-1^, 1SD, *n* = 3), with room-dried samples showing the same mean but slightly higher variability (12 ± 1.08 ng g^-1^, 1SD, *n* = 3). In the unrinsed category, oven-dried inner foliage samples exhibited slightly elevated Hg levels (13 ± 1.6 ng g^-1^, 1SD, *n* = 3) compared to both freeze-dried (12 ± 0.97 ng g^-1^, 1SD, *n* = 3) and room-dried samples (12 ± 1.4 ng g^-1^, 1SD, *n* = 3).

For outer foliage samples, the rinsed category showed a slight increasing trend from freeze-dried (8.2 ± 2.6 ng g^-1^, 1SD, *n* = 3) to oven-dried (8.7 ± 1.9 ng g^-1^, 1SD, *n* = 3) to room-dried samples (8.8 ± 0.57 ng g^-1^, 1SD, *n* = 3). A more pronounced increase was observed in unrinsed outer foliage, with room-dried samples (10 ± 3.6 ng g^-1^, 1SD, *n* = 3) containing substantially higher Hg concentrations than both freeze-dried (8.2 ± 1.1 ng g^-1^, 1SD, *n* = 3) and oven-dried samples (8.6 ± 1.6 ng g^-1^, 1SD, *n* = 3). Upper foliage samples in the rinsed category displayed an increasing trend across drying methods, with the highest concentrations in room-dried samples (9.0 ± 1.3 ng g^-1^, 1SD, *n* = 3), followed by oven-dried (8.4 ± 0.93 ng g^-1^, 1SD, *n* = 3) and freeze-dried samples (8.0 ± 1.9 ng g^-1^, 1SD, *n* = 3). Similarly, for unrinsed upper foliage, room-dried samples contained the highest Hg levels (8.7 ± 2.1 ng g^-1^, 1SD, *n* = 3), while freeze-dried (8.4 ± 0.84 ng g^-1^, 1SD, *n* = 3) and oven-dried samples (8.3 ± 0.39 ng g^-1^, 1SD, *n* = 3) showed comparable concentrations. It is noteworthy here that residual moisture content was consistently higher in room-dried samples compared to oven- and freeze-dried samples; full moisture data and statistical comparisons are provided in the Online Resource 1. Despite these observed differences in Hg concentrations across canopy positions and drying methods, statistical analysis revealed no significant differences (*p* > 0.05, Table S1, Online Resource 1). The observed variability is largely due to natural heterogeneity within the canopy rather than any systematic effect of the drying method used. The effect of different drying on vertical foliar Hg isotopic signatures were not investigated in this work.

### Effect of sample rinsing

Rinsing process did not result in a substantial decrease in Hg concentration in Ljubljana, indicating a minimal presence of water-leachable Hg on the foliar surfaces at this site, as shown in Table S1 (Online Resource 1). For inner foliage, mean Hg concentrations in freeze-dried samples were identical for rinsed and unrinsed sets (12 ± 0.91 ng g^-^^1^, 1SD, *n* = 3 and 12 ± 0.97 ng g^-^^1^, 1SD, *n* = 3, respectively). Similar results were observed for oven-dried (rinsed: 12 ± 0.35 ng g^-^^1^; unrinsed: 13 ± 1.6 ng g^-^^1^) and room-dried samples (rinsed: 12 ± 1.08 ng g^-^^1^; unrinsed: 12 ± 1.4 ng g^-^^1^). Outer foliage samples also exhibited close agreement between rinsed and unrinsed treatments, with freeze-dried (rinsed: 8.2 ± 2.6 ng g^-^^1^; unrinsed: 8.2 ± 1.1 ng g^-^^1^), oven-dried (rinsed: 8.7 ± 1.9 ng g^-^^1^; unrinsed: 8.6 ± 1.6 ng g^-^^1^), and room-dried samples (rinsed: 8.8 ± 0.57 ng g^-^^1^; unrinsed: 10 ± 3.6 ng g^-^^1^) all showing overlapping ranges. For upper foliage, Hg concentrations were also comparable between rinsed and unrinsed sets across all drying categories, with freeze-dried (rinsed: 8.0 ± 1.9 ng g^-^^1^; unrinsed: 8.4 ± 0.84 ng g^-^^1^), oven-dried (rinsed: 8.4 ± 0.93 ng g^-^^1^; unrinsed: 8.3 ± 0.39 ng g^-^^1^), and room-dried samples (rinsed: 9.0 ± 1.3 ng g^-^^1^; unrinsed: 8.7 ± 2.1 ng g^-^^1^) showing differences in means generally within 1 ng g^-^^1^. These differences however, were not statistically significant (*p* = 0.05) for individual pre-treatment test (Table S1. Online Resource 1) indicating that, under the conditions tested, rinsing did not systematically alter measured Hg concentrations in foliage from Ljubljana. The small differences observed are likely due to natural variability within the canopy and not to the rinsing procedure itself. Notably, rinsing before drying led to a modest yet statistically significant increase in residual moisture content for all drying methods, with the largest effect observed in room- and oven-dried samples. Comprehensive data and statistical analyses are available in Online Resource 1.

The influence of sample rinsing on the foliar Hg isotopic signatures within the tree canopy was also investigated, as the isotopic signatures of Hg deposited on the surface of foliage may display distinct characteristics from those assimilated within the leaf structure, given that both forms undergo different fractionation processes. Surface-bound Hg is often influenced by atmospheric deposition and can reflect the isotopic composition of environmental sources, while internally assimilated Hg is subject to biological and chemical processes within the foliage, leading to unique isotopic signatures (Demers et al., 2013; Wang et al., 2022; Yuan et al., 2019). This difference underscores the importance of distinguishing between these two forms when analyzing foliar Hg isotopes, as it can significantly affect interpretations of Hg dynamics and sources in ecosystems.

**Fig. 3:**
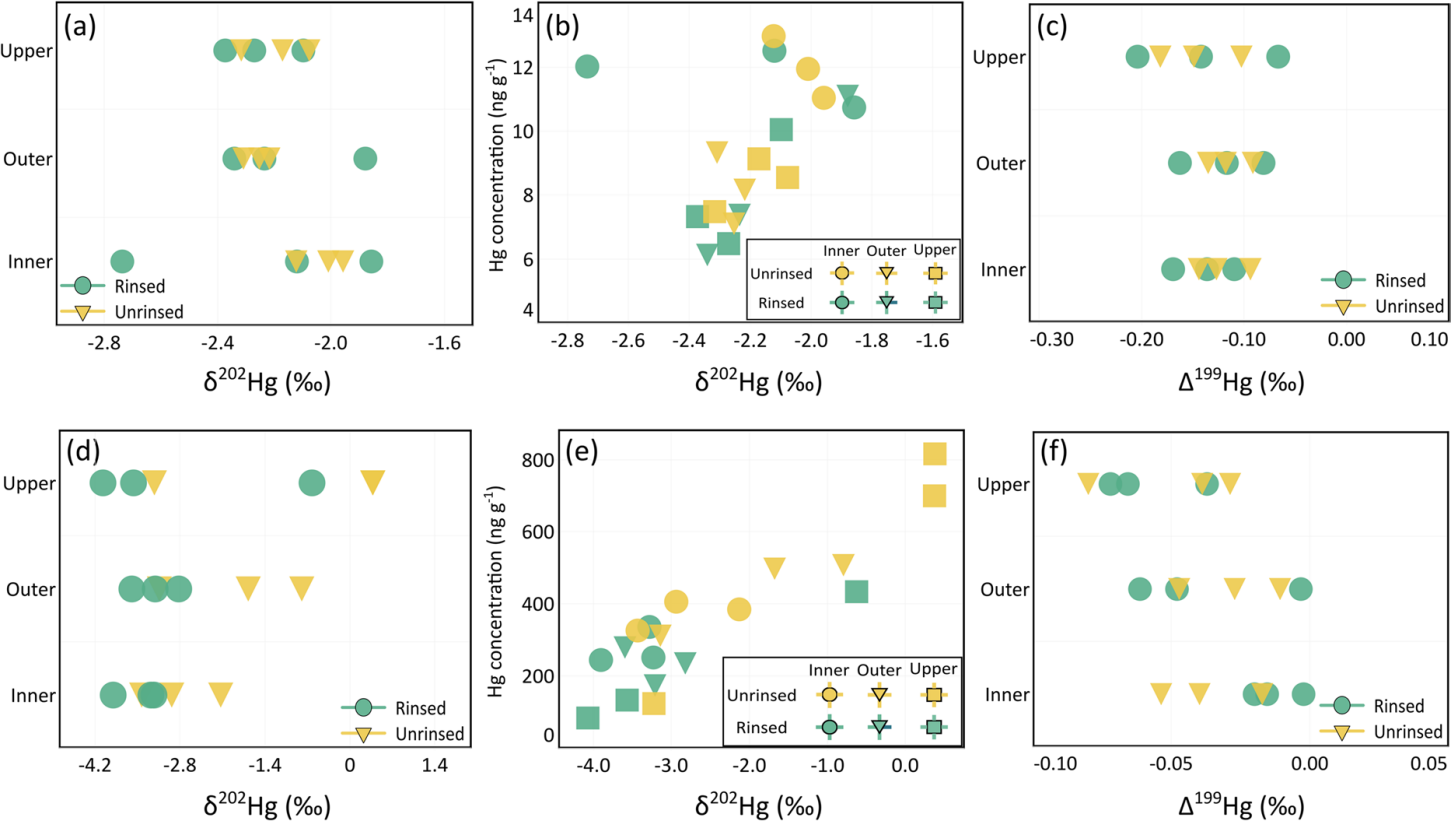
Effect of sample rinsing on foliar Hg isotopic signatures from Ljubljana and Idrija. Vertical distribution of δ^20^^2^Hg isotopic signatures within the tree crown for Ljubljana (**a**) and Idrija (**d**). δ^2^^0^^2^Hg isotopic signatures shown as a function of Hg concentration for Ljubljana (**b**) and Idrija (**e**). Effect of sample rinsing on the vertical distribution of Δ.^199^Hg isotope signatures within the tree crown in Ljubljana (**c**) and Idrija (**f**)

In Ljubljana, rinsed foliar δ^202^Hg values averaged -2.2 ± 0.27‰ (1SD, *n* = 9), whereas unrinsed foliar δ^202^Hg values averaged -2.2 ± 0.13‰ (1SD, *n* = 9). The sample rinsing process led to negligible changes in MDF values across all three crown positions (Fig. 3b). However, the unrinsed foliage sample from the inner part of the tree crown, which showed higher Hg concentration, displayed a relatively less negative MDF signature. This less negative MDF signature may reflect a minor contribution of PBM adhering to leaf surfaces, which can carry Hg with distinct isotopic compositions. These particles, although present in small amounts, can influence isotopic measurements due to their enriched heavier isotopic signatures. When the foliage samples were rinsed, a small change in the foliar Hg concentration was observed, with average Hg concentrations for unrinsed inner canopy samples (12 ± 0.97 ng g^-^^1^, *n* = 3) slightly higher than rinsed samples (12 ± 0.91 ng g^-^^1^, *n* = 3). However, this difference was statistically insignificant (*p* > 0.05). Despite this negligible change in Hg concentration, the rinsing process led to a broader range of MDF signatures among individual samples. This suggests that while rinsing did not substantially alter the overall Hg concentration, it selectively removed surface-bound Hg with distinct isotopic compositions in a non-uniform manner, likely due to differences in leaf surface characteristics or particle adhesion. Additionally, the lack of change in the Hg concentration following sample rinsing suggests that Hg in foliage samples from Ljubljana mainly originated as a result of foliar Hg^0^ uptake and Hg deposition on foliar surfaces was negligible, under the assumption that rinsing effectively removes loosely bound surface Hg. As such, significant changes in Hg isotope ratios are expected only when there is a corresponding change in Hg concentration resulting from the addition or removal of isotopically distinct Hg. If the Hg content remains constant, notable shifts in Hg isotope ratios are unlikely to occur. The influence of rinsing on the MIF signatures in foliage samples from all positions on the tree crown was also not discernible (Fig. 3c).

### Vertical Hg distribution within tree crown in Idrija

Across all the pre-treatment tests on Idrija samples, higher Hg concentrations were observed in the outer foliage samples compared to the inner and upper samples, as indicated by the variation in in-canopy foliar Hg concentrations. The mean Hg concentration (averaged across the drying and rinsing categories) of the outer foliage samples was 388 ± 209 ng g^-^^1^ (1SD, *n* = 18), whereas the inner and upper crown foliage samples had mean concentrations of 336 ± 88 ng g^-^^1^ (1SD, *n* = 18) and 241 ± 207 ng g^-^^1^ (1SD, *n* = 18), respectively. The observed elevation in Hg concentrations in outer foliage samples at the Idrija site can be attributed to a combination of leaf physiological traits and environmental exposure dynamics. Outer crown leaves exhibit a lower specific leaf area (SLA) compared to inner crown leaves, as demonstrated in *Acer rubrum* by (Laacouri et al., 2013). This anatomical distinction reflects thicker outer foliage with greater biomass per unit surface area, enabling higher Hg storage capacity on a per-area basis despite similar mass-based concentrations. Enhanced stomatal conductance in sun-exposed outer leaves further facilitates uptake of Hg⁰ through stomatal diffusion, a process strongly correlated with photosynthetic activity (Herrick et al., 2004; Morecroft & Roberts, 1999). However, the pronounced Hg enrichment in outer foliage at Idrija, proximate to historical smelting activity, likely arises synergistically from PBM deposition. Outer leaves, positioned at the canopy periphery, experience greater airflow turbulence and intercept resuspended soil particulates enriched in Hg from legacy contamination (Tomiyasu et al., 2017). The interplay of these factors explains the vertical Hg gradient observed in Idrija’s tree crowns. The influence of sample drying and rinsing methods on the vertical distribution of foliar Hg concentrations is discussed in the following sections.

### Effect of sample drying

Hg concentrations in Idrija samples demonstrated distinct patterns when comparing the different drying methods for each foliage position and sample pre-treatment category. For inner foliage samples in the rinsed category, freeze-dried samples exhibited moderate Hg concentrations (280 ± 50 ng g^-^^1^, 1SD, *n* = 3), closely matched by oven-dried samples (280 ± 35 ng g^-^^1^, 1SD, *n* = 3), while room-dried samples showed higher mean concentrations (310 ± 88 ng g^-^^1^, 1SD, *n* = 3). In the unrinsed category, room-dried inner samples yielded the highest Hg levels (430 ± 150 ng g^-^^1^, 1SD, *n* = 3), followed by freeze-dried (370 ± 41 ng g^-^^1^, 1SD, *n* = 3) and oven-dried samples (370 ± 74 ng g^-^^1^, 1SD, *n* = 3). For outer foliage samples in the rinsed category, Hg concentrations increased from freeze-dried (230 ± 53 ng g^-^^1^, 1SD, *n* = 3) to oven-dried (260 ± 58 ng g^-^^1^, 1SD, *n* = 3) to room-dried samples (340 ± 210 ng g^-^^1^, 1SD, *n* = 3). In the unrinsed category, room-dried outer samples exhibited substantially higher Hg concentrations (690 ± 320 ng g^-^^1^, 1SD, *n* = 3) compared to freeze-dried (440 ± 110 ng g^-^^1^, 1SD, *n* = 3) and oven-dried samples (390 ± 140 ng g^-^^1^, 1SD, *n* = 3). Upper foliage samples in the rinsed category displayed the highest Hg levels in freeze-dried samples (220 ± 190 ng g^-^^1^, 1SD, *n* = 3), followed by room-dried (160 ± 70 ng g^-^^1^, 1SD, *n* = 3) and oven-dried samples (120 ± 21 ng g^-^^1^, 1SD, *n* = 3). Similarly, for unrinsed upper foliage, freeze-dried samples contained the highest Hg concentrations (550 ± 370 ng g^-^^1^, 1SD, *n* = 3), with room-dried (210 ± 60 ng g^-^^1^, 1SD, *n* = 3) and oven-dried samples (190 ± 62 ng g^-^^1^, 1SD, *n* = 3) showing lower values.

Statistical analysis showed no significant differences in foliar Hg concentrations between drying methods for any foliage position or treatment category in Idrija (all *p* > 0.05; see Table S2). This indicates that the drying method generally did not affect Hg concentrations across all foliage types in Idrija. The variability observed among samples is likely due to high heterogeneity in PBM on foliar surfaces, a result of historical cinnabar contamination in Idrija. This sample heterogeneity, known as the nugget effect, drives pronounced differences in Hg measurements for some samples, rather than the drying method itself (Kocman et al., 2011). Conversely, room-dried foliage consistently retained higher residual moisture content than oven- or freeze-dried samples, for both rinsed and unrinsed treatments. The differences were statistically significant for comparisons involving room drying, but not between oven and freeze drying. Full results and statistical details are provided in Online Resource 1.

### Effect of sample rinsing

In Idrija, rinsing significantly reduced Hg concentrations in foliage samples, indicating that a substantial portion of Hg was present on the foliar surfaces (Table S2, Online Resource 1). For freeze-dried samples, rinsed inner foliage showed a mean Hg concentration of 280 ± 50 ng g^-^^1^ (1SD, *n* = 3), compared to 370 ± 41 ng g^-^^1^ (1SD, *n* = 3) in unrinsed samples (*p* = 0.001). Outer foliage displayed a similar pattern, with rinsed samples at 230 ± 53 ng g^-^^1^ (1SD, *n* = 3) and unrinsed at 440 ± 110 ng g^-^^1^ (1SD, *n* = 3; *p* = 0.008). Upper foliage also demonstrated a marked decrease after rinsing (rinsed: 220 ± 190 ng g^-^^1^, 1SD, *n* = 3; unrinsed: 550 ± 370 ng g^-1^, 1SD, *n* = 3; *p* = 0.018). Oven-dried samples followed the same trend. Rinsed inner foliage averaged 280 ± 35 ng g^-^^1^ (1SD, *n* = 3), while unrinsed samples had 370 ± 74 ng g^-^^1^ (1SD, *n* = 3). For outer foliage, rinsed samples were 260 ± 58 ng g^-^^1^ (1SD, *n* = 3) and unrinsed 390 ± 140 ng g^-^^1^(1SD, *n* = 3). Upper foliage showed 120 ± 21 ng g^-^^1^ (1SD, *n* = 3) for rinsed and 190 ± 62 ng g^-^^1^ (1SD, *n* = 3) for unrinsed samples (*p* = 0.087). These differences were not statistically significant for oven-dried samples. Room-dried samples exhibited the largest absolute differences. Rinsed inner foliage contained 310 ± 88 ng g^-1^ (1SD, *n* = 3), while unrinsed samples reached 430 ± 150 ng g^-^^1^ (1SD, *n* = 3; *p* = 0.043). For outer foliage, rinsed samples had 340 ± 210 ng g^-^^1^ (1SD, *n* = 3) versus 690 ± 320 ng g^-^^1^ (1SD, *n* = 3) in unrinsed samples (*p* = 0.025). Upper foliage was 160 ± 70 ng g^-^^1^ (1SD, *n* = 3) for rinsed and 210 ± 60 ng g^-^^1^ (1SD, *n* = 3) for unrinsed samples. These results demonstrate that rinsing significantly reduces measured foliar Hg concentrations in Idrija, regardless of canopy position. Across the nine paired measurements covering all three canopy positions and three drying methods, rinsing removed 37 ± 13% (1SD, *n*= 9) of foliar Hg, corresponding to a mean absolute loss of 160 ± 112 ng g^-^^1^ (1SD, *n* = 9). The substantial differences between rinsed and unrinsed samples potentially highlight the influence of rinsing on reducing surface-bound Hg in foliage samples from this historically contaminated site. In contrast to Ljubljana, rinsing had little effect on residual moisture content in Idrija samples across all drying methods, with only minor and statistically insignificant changes observed for room-, oven-, and freeze-dried samples. These results indicate that rinsing did not introduce appreciable additional water in Idrija foliage, regardless of drying protocol. Full data and statistical comparisons are provided in Online Resource 1.

**Fig. 4:**
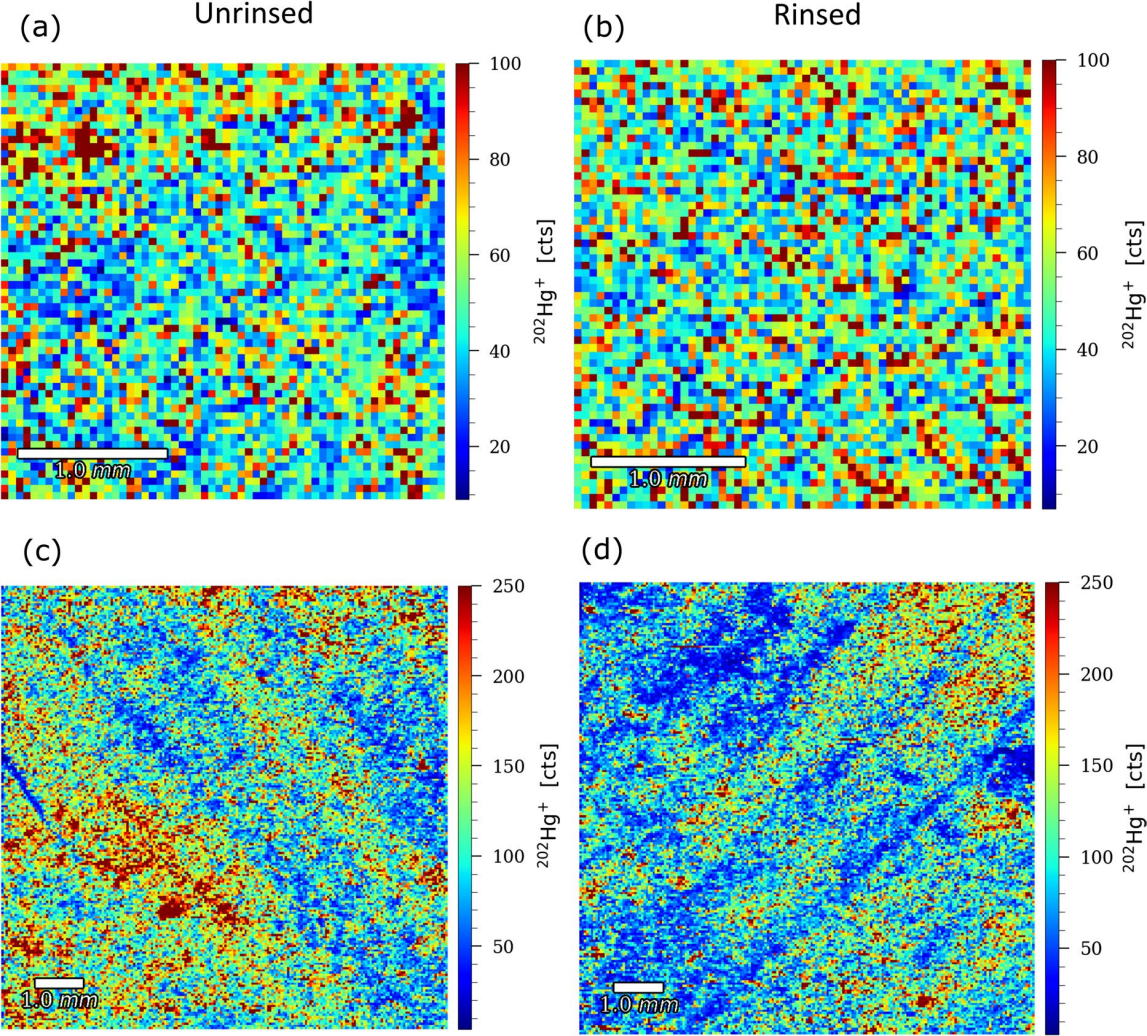
Influence of sample rinsing on the spatial distribution of Hg counts per pixel in unrinsed and rinsed foliage samples from Idrija for (**a**, **b**) 3 mm x 3 mm and (**c**, **d**) 10 mm × 10 mm ablated areas on foliage samples

To further elucidate the effect of sample rinsing on foliar Hg content, we performed a spatial analysis of changes in foliar Hg distribution in Idrija. LA-ICP-MS images displaying the relative distribution of foliar Hg are shown in Fig. 4. The highest pixel concentration of Hg is shown by the intensity of the red colour. The effect of sample rinsing on the spatial distribution of Hg in foliage samples was demonstrated by the difference in Hg counts per pixel for a surface area of 9 mm^2^ between rinsed and unrinsed foliage sample (Fig. 4a, b; rinsed: 57, unrinsed: 58), corresponding to a 2% reduction in Hg counts per pixel for rinsed samples. Moreover, the ablation of a wider surface area (100 mm^2^) resulted in a substantial difference in Hg counts per pixel for the ablated sections of the foliage surface (Fig. 4c, d; rinsed: 115, unrinsed: 132), corresponding to a 13% reduction in Hg counts per pixel for rinsed samples. Although natural variation in Hg distribution in foliage samples is anticipated, the presence of Hg hotspots on foliage surfaces is evident and may indicate the presence of PBM on foliar surfaces. By contrast, bulk Hg analyses of the Idrija foliage samples showed an average rinsing‐induced Hg loss of 37 ± 13% (1SD, *n* = 9), substantially higher than the 2% and 13% reductions detected by LA-ICP-MS at 9 and 100 mm^2^, respectively. The discrepancy between the pronounced rinsing effect observed in bulk Hg measurements and the comparatively modest reductions detected by LA-ICP-MS can be attributed to differences in sample size and analytical scale. Bulk analyses were conducted on large aliquots derived from 200 g of fresh foliage, increasing the likelihood of capturing PBM or surface hotspots within the sample. In contrast, LA-ICP-MS measurements targeted much smaller areas (3 × 3 mm and 10 × 10 mm) on a single leaf sample, greatly reducing the probability of including such particles or localized Hg-rich regions. As a result, the LA-ICP-MS data are less susceptible to the “nugget effect” and may underestimate the magnitude of rinsing-induced Hg removal observed in bulk analyses. This methodological difference likely explains the observed discrepancies in the rinsing effect between the two approaches.

Hg isotopic analysis of freeze-dried foliage samples revealed significant variations in their MDF signatures (Fig. 3d), whereas no significant MIF was observed (Fig. 3e). The large magnitude of variation in MDF values and the smaller magnitude of variation in MIF values in Idrija is mainly due to the highly heterogeneous nature of the local area with elevated Hg presence (Božič et al., 2023). This heterogeneity reflects the spatially variable distribution of legacy Hg contamination (e.g., cinnabar particles) from historical mining activities in Idrija. For rinsed samples, the removal of PBM via washing reduced surface-derived Hg contributions, resulting in MDF signatures dominated by internal foliar Hg pools assimilated via stomatal uptake of gaseous Hg^0^. Consequently, the remaining isotopic variability in rinsed samples reflects intrinsic biological processes (e.g., Hg^0^ assimilation, re-emission) rather than external environmental heterogeneity. The effect of sample rinsing on the foliar MDF signatures was particularly observed for the outer and upper foliage samples, indicating that the outer and upper positions of the tree crown received a greater deposition of PBM onto the foliar surface than the inner parts (Fig. 3d). This is in line with the higher Hg concentrations observed in the outer and upper positions of the unrinsed freeze-dried foliage samples than in the inner crown position (Fig. 2b). Subsequently, the rinsing process removed significant amounts of Hg from the foliage samples, (mean Hg difference = 160 ± 112 ng g^-^^1^, 1 SD, *n* = 9), equivalent to a 37 ± 13% reduction relative to the unrinsed foliage (paired t-test, *p* = 0.003), causing the MDF signatures to shift to more negative values.

Rinsed foliage samples from Idrija displayed a δ^202^Hg value of -3.2 ± 1.0‰ (1SD, *n* = 9), while unrinsed samples had a δ^202^Hg of -1.9 ± 1.5‰ (1SD, *n* = 9), indicating that sample rinsing removed significant foliar Hg enriched in heavier isotopes. This difference reflects the distinct environmental conditions and contamination histories of these sites. The Idrija region, impacted by centuries of Hg mining and smelting activities, has created a highly heterogeneous isotopic reservoir with δ^20^^2^Hg values ranging from -1.35‰ to 0.46‰ in cinnabar ores and associated materials (Božič et al., 2023). High soil Hg concentrations and substantial Hg⁰ emissions from contaminated surfaces promote the uptake of isotopically lighter Hg by vegetation through stomatal pathways. In addition, PBM enriched in heavier isotopes contributes to isotopic variability when deposited on leaf surfaces. In contrast, Ljubljana's urban environment is dominated by atmospheric Hg⁰ uptake with minimal PBM deposition, resulting in less negative δ^2^^0^^2^Hg values and lower variability in rinsed samples.

## Conclusions

The dynamics of Hg uptake and accumulation in forest ecosystems are complex and are therefore, influenced by several factors including canopy structure, environmental settings, and methodological approaches. This study systematically evaluated the effects of sampling and pre-treatment methods, including drying and rinsing, on foliar Hg concentrations and the effect of sample rinsing on foliar Hg isotopic signatures, providing insights into best practices for future research.

The findings demonstrate that drying methods (freeze-drying, oven-drying, and room-drying) do not significantly alter foliar Hg concentrations in low-Hg environments, supporting cross-study comparability. However, freeze-drying is recommended for isotopic studies due to its ability to minimize Hg volatilization and preserve isotopic integrity. Tailoring protocols to site-specific Hg sources is crucial, as these sources influence both Hg concentrations and isotopic signatures. Rinsing is recommended when assessing internal plant Hg cycling in regions with elevated PBM contamination, whereas it may be omitted if the total foliage-associated Hg pool, including surface-bound fractions, is of interest. A standardized rinsing protocol using Milli-Q water with multiple submersions ensures the removal of PBM while minimizing leaching of internal foliar Hg. Bioimaging results further confirmed the presence of Hg hotspots on foliage surfaces, highlighting the importance of rinsing in regions with high PBM deposition. Spatial variability in Hg deposition within tree canopies was evident, with inner canopy foliage exhibiting higher Hg concentrations due to reduced solar exposure and increased throughfall deposition. Sampling strategies should account for canopy position (inner, outer, upper) to capture this variability accurately. Consistent reporting of metadata on canopy position and environmental conditions is critical for facilitating cross-study comparisons.

This study underscores the need for harmonized protocols for quantifying foliar Hg concentrations and isotopic signatures. Standardizing sample preparation methods will enhance measurement accuracy across diverse studies and locations while improving modelling results of forest-atmosphere Hg exchanges. Future research should investigate inter-species differences, seasonal variability in foliar Hg dynamics, and the effects of drying methods on isotopic fractionation to refine methodological consistency further and deepen understanding of Hg cycling in forest ecosystems.

## Supplementary Information

Below is the link to the electronic supplementary material.

Supplementary file1 (DOCX 5810 KB)

## Data Availability

The authors declare that the data supporting the findings of this study are available within the paper and its Supplementary Information files. Should any raw data files be needed in another format they are available from the corresponding author upon reasonable request.
