# Supplementary material for: Evaluation of sampling and sample preparation methodologies for determination of mercury concentrations and stable mercury isotopes in foliage samples
Source: Environ Monit Assess. 2025 Jul 4;197(8):856. doi: 10.1007/s10661-025-14318-6 (PMC12226646; doi:10.1007/s10661-025-14318-6)
Supplement: Supplementary file 1 — Supplementary file1 (DOCX 5810 KB) [file 10661_2025_14318_MOESM1_ESM.docx]

Journal: Environmental Monitoring and Assessment

Title: Evaluation of sampling and sample preparation methodologies for determination of mercury concentrations and stable mercury isotopes in foliage samples.

Authors: Saeed Waqar Ali^a,b^, Dominik Božič^a,b^, Sreekanth Vijayakumaran Nair^a,b^, Igor Živković^a,b^, Teodor-Daniel Andron^a,b^, Stefan Marković^a^, Marta Jagodic Hudobivnik^a^, Milena Horvat^a,b^, David Kocman^a∗^

Affiliations: ^a∗^Department of Environmental Sciences, Jožef Stefan Institute, 1000, Ljubljana, Slovenia, ^b^Jožef Stefan International Postgraduate School, 1000, Ljubljana, Slovenia.

Corresponding author: [david.kocman@ijs.si](mailto:david.kocman@ijs.si)

Supplementary information


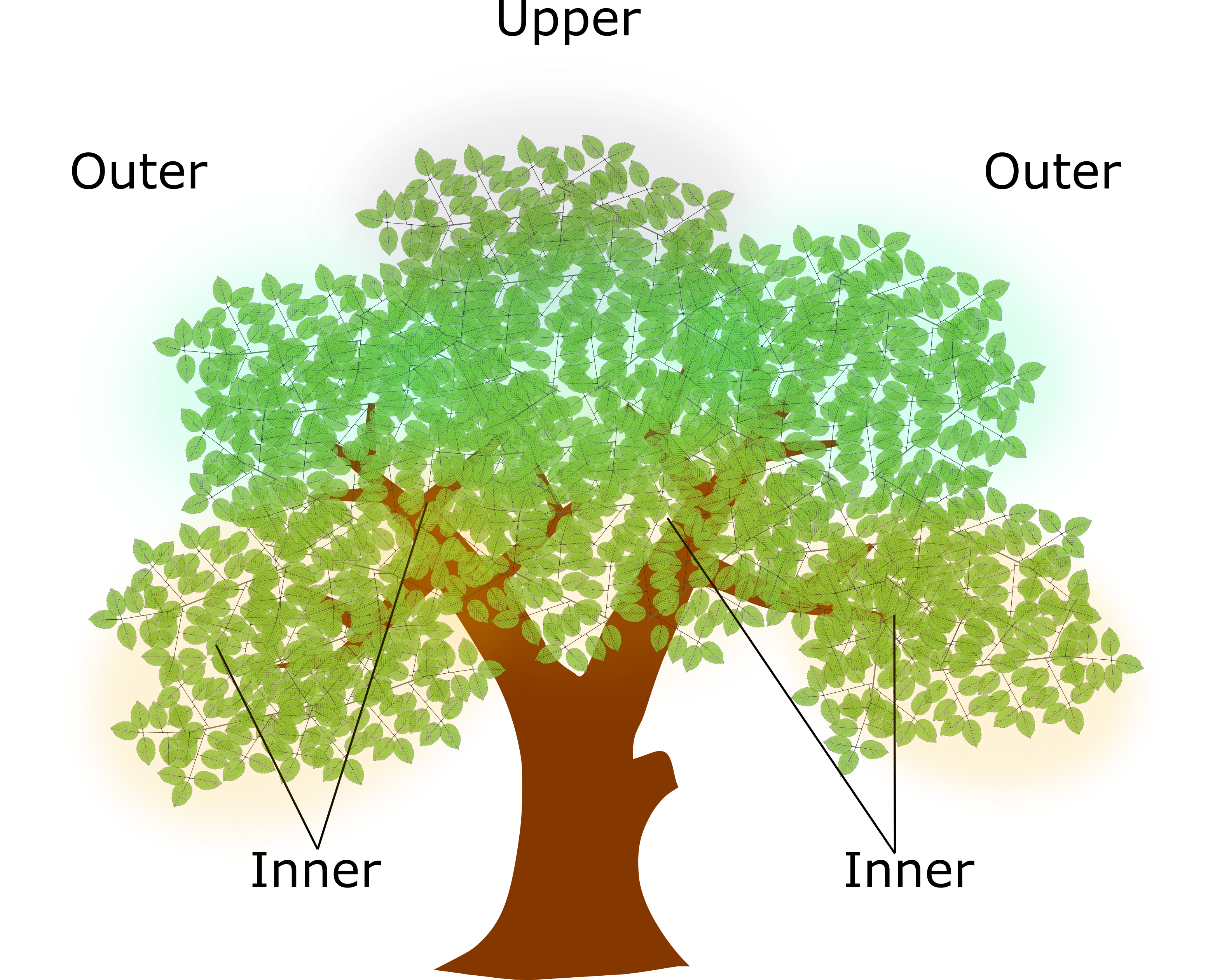


**Fig.** S1 Schematic representation of foliage sampling from three distinct locations on the tree crown.

Table S1. Statistically significant differences in sample pre-treatment tests for Ljubljana samples are presented. A two-tailed paired t-test was performed to evaluate the effects of rinsing and drying on foliar Hg concentrations, and the numbers in the table represent the corresponding p-values; values shown in bold indicate statistically significant differences (p < 0.05).

|  | **Factor** | **Comparison** | **Unrinsed** | **Rinsed** | **Rinsed vs Unrinsed** |
| --- | --- | --- | --- | --- | --- |
| Rinsing effect | Room dried | Inner |  |  | 0.480 |
|  | Room dried | Outer |  |  | 0.202 |
|  | Room dried | Upper |  |  | 0.496 |
|  |  |  |  |  |  |
|  | Oven Dried | Inner |  |  | 0.181 |
|  | Oven Dried | Outer |  |  | 0.436 |
|  | Oven Dried | Upper |  |  | 0.321 |
|  |  |  |  |  |  |
|  | Freeze Dried | Inner |  |  | 0.328 |
|  | Freeze Dried | Outer |  |  | 0.500 |
|  | Freeze Dried | Upper |  |  | 0.333 |
| Crown position | Room dried | Inner vs Outer | 0.553 | **0.002** |  |
|  | Room dried | Inner vs Upper | 0.079 | **0.004** |  |
|  | Room dried | Outer vs Upper | 0.513 | 0.740 |  |
|  |  |  |  |  |  |
|  | Oven Dried | Inner vs Outer | **0.021** | **0.002** |  |
|  | Oven Dried | Inner vs Upper | **0.027** | **0.000** |  |
|  | Oven Dried | Outer vs Upper | 0.855 | 0.719 |  |
|  |  |  |  |  |  |
|  | Freeze Dried | Inner vs Outer | **0.012** | **0.013** |  |
|  | Freeze Dried | Inner vs Upper | **0.009** | **0.002** |  |
|  | Freeze Dried | Outer vs Upper | 0.827 | 0.844 |  |
| Drying effects | Inner | Room vs Oven | 0.394 | 0.826 |  |
|  | Inner | Room vs Freeze | 0.849 | 0.658 |  |
|  | Inner | Oven vs Freeze | 0.285 | 0.395 |  |
|  |  |  |  |  |  |
|  | Outer | Room vs Oven | 0.427 | 0.703 |  |
|  | Outer | Room vs Freeze | 0.352 | 0.865 |  |
|  | Outer | Oven vs Freeze | 0.743 | 0.753 |  |
|  |  |  |  |  |  |
|  | Upper | Room vs Oven | 0.819 | 0.417 |  |
|  | Upper | Room vs Freeze | 0.656 | 0.303 |  |
|  | Upper | Oven vs Freeze | 0.531 | 0.634 |  |

Table S2. Statistically significant differences between sample pre-treatment tests for Idrija samples. A two-tailed paired t-test was performed to evaluate the effects of rinsing and drying on foliar Hg concentrations, and the numbers in the table represent the corresponding p-values; values shown in bold indicate statistically significant differences (p < 0.05).

|  | **Factor** | **Comparison** | **Unrinsed** | **Rinsed** | **Rinsed vs Unrinsed** |
| --- | --- | --- | --- | --- | --- |
| Rinsing effect | Room dried | Inner |  |  | **0.043** |
|  | Room dried | Outer |  |  | **0.025** |
|  | Room dried | Upper |  |  | 0.083 |
|  |  |  |  |  |  |
|  | Oven Dried | Inner |  |  | 0.071 |
|  | Oven Dried | Outer |  |  | 0.100 |
|  | Oven Dried | Upper |  |  | 0.087 |
|  |  |  |  |  |  |
|  | Freeze Dried | Inner |  |  | **0.001** |
|  | Freeze Dried | Outer |  |  | **0.008** |
|  | Freeze Dried | Upper |  |  | **0.018** |
| Crown position | Room dried | Inner vs Outer | 0.295 | 0.820 |  |
|  | Room dried | Inner vs Upper | 0.105 | 0.087 |  |
|  | Room dried | Outer vs Upper | 0.115 | 0.273 |  |
|  |  |  |  |  |  |
|  | Oven Dried | Inner vs Outer | 0.843 | 0.669 |  |
|  | Oven Dried | Inner vs Upper | **0.032** | **0.005** |  |
|  | Oven Dried | Outer vs Upper | 0.111 | **0.043** |  |
|  |  |  |  |  |  |
|  | Freeze Dried | Inner vs Outer | 0.411 | 0.322 |  |
|  | Freeze Dried | Inner vs Upper | 0.502 | 0.641 |  |
|  | Freeze Dried | Outer vs Upper | 0.671 | 0.922 |  |
| Drying effects | Inner | Room vs Oven | 0.554 | 0.596 |  |
|  | Inner | Room vs Freeze | 0.577 | 0.644 |  |
|  | Inner | Oven vs Freeze | 0.904 | 0.938 |  |
|  |  |  |  |  |  |
|  | Outer | Room vs Oven | 0.235 | 0.565 |  |
|  | Outer | Room vs Freeze | 0.305 | 0.462 |  |
|  | Outer | Oven vs Freeze | 0.629 | 0.592 |  |
|  |  |  |  |  |  |
|  | Upper | Room vs Oven | 0.636 | 0.417 |  |
|  | Upper | Room vs Freeze | 0.252 | 0.658 |  |
|  | Upper | Oven vs Freeze | 0.231 | 0.462 |  |

Table S3. Descriptive statistics of Hg concentrations (ng g^-1^) from the pre-treatment tests on foliage samples from Ljubljana and Idrija.

|  |  | **Freeze Dried** | | | | | | **Oven Dried** | | | | | | **Room Dried** | | | | | |
| --- | --- | --- | --- | --- | --- | --- | --- | --- | --- | --- | --- | --- | --- | --- | --- | --- | --- | --- | --- |
|  |  | **Rinsed** | | | **Unrinsed** | | | **Rinsed** | | | **Unrinsed** | | | **Rinsed** | | | **Unrinsed** | | |
| **Site** |  | **Inner** | **Outer** | **Upper** | **Inner** | **Outer** | **Upper** | **Inner** | **Outer** | **Upper** | **Inner** | **Outer** | **Upper** | **Inner** | **Outer** | **Upper** | **Inner** | **Outer** | **Upper** |
| Ljubljana | Average | 12 | 8.2 | 8.0 | 12 | 8.2 | 8.4 | 12 | 8.7 | 8.4 | 13 | 8.6 | 8.3 | 12 | 8.8 | 9.0 | 12 | 10 | 8.7 |
|  | Std dev | 0.91 | 2.6 | 1.9 | 0.97 | 1.1 | 0.84 | 0.35 | 1.9 | 0.9 | 1.6 | 1.6 | 0.39 | 1.1 | 0.57 | 1.3 | 1.4 | 3.6 | 2.1 |
|  | Sample 1 | 12 | 11 | 10 | 13 | 8.2 | 9.1 | 12 | 9.4 | 9.3 | 1 | 9.9 | 8.7 | 11 | 9.0 | 10 | 14 | 15 | 11 |
|  | Sample 2 | 10 | 7.4 | 6.5 | 11 | 9.3 | 8.6 | 12 | 10 | 8.3 | 12 | 9.1 | 8.3 | 12 | 9.1 | 7.9 | 12.0 | 9.8 | 8.0 |
|  | Sample 3 | 12 | 6.1 | 7.3 | 12 | 7.1 | 7.5 | 13 | 6.5 | 7.5 | 12 | 7.0 | 7.9 | 13 | 8.1 | 8.6 | 11 | 7.7 | 7.1 |
| Idrija | Average | 280 | 230 | 220 | 370 | 440 | 550 | 280 | 260 | 120 | 370 | 390 | 190 | 310 | 340 | 160 | 430 | 690 | 210 |
|  | Std dev | 50 | 53 | 190 | 41 | 110 | 370 | 35 | 58 | 21 | 74 | 140 | 62 | 88 | 210 | 70 | 150 | 320 | 60 |
|  | Sample 1 | 250 | 280 | 130 | 390 | 310 | 120 | 240 | 310 | 130 | 370 | 380 | 140 | 290 | 220 | 160 | 450 | 410 | 170 |
|  | Sample 2 | 250 | 240 | 80 | 330 | 500 | 700 | 280 | 260 | 100 | 290 | 530 | 200 | 230 | 600 | 90 | 270 | 1000 | 180 |
|  | Sample 3 | 340 | 170 | 430 | 400 | 510 | 820 | 310 | 200 | 130 | 400 | 250 | 250 | 400 | 210 | 230 | 570 | 610 | 280 |

Table S4. Hg isotope values for δ^202^Hg (‰) and ∆^199^Hg (‰) along with Hg concentrations (ng g^-1^) for freeze-dried samples from Ljubljana and Idrija categorized based on crown position, rinsing procedure and sampling site.

| SN | **Sample** | **Crown position** | **Rinsing** | **Site** | **δ^202^Hg (‰)** | **∆^199^Hg (‰)** | **∆^200^Hg** | **∆^201^Hg** | **∆^204^Hg** | **Hg concentration (ng g^-1^)** |
| --- | --- | --- | --- | --- | --- | --- | --- | --- | --- | --- |
| 1 | 1 | Inner | Rinsed | Ljubljana | -2.74 | -0.12 | 0.02 | -0.10 | -0.01 | 12 |
| 2 | 2 | Inner | Rinsed | Ljubljana | -1.86 | -0.11 | 0.02 | -0.13 | 0.00 | 11 |
| 3 | 3 | Inner | Rinsed | Ljubljana | -2.12 | -0.14 | 0.04 | -0.13 | 0.03 | 12 |
| 4 | 1 | Outer | Rinsed | Ljubljana | -1.88 | -0.12 | 0.08 | -0.13 | -0.07 | 11 |
| 5 | 2 | Outer | Rinsed | Ljubljana | -2.24 | -0.08 | 0.03 | -0.10 | 0.02 | 7.4 |
| 6 | 3 | Outer | Rinsed | Ljubljana | -2.34 | -0.16 | 0.00 | -0.14 | -0.03 | 6.1 |
| 7 | 1 | Upper | Rinsed | Ljubljana | -2.1 | -0.07 | 0.00 | -0.10 | 0.00 | 10 |
| 8 | 2 | Upper | Rinsed | Ljubljana | -2.27 | -0.14 | 0.00 | -0.17 | -0.02 | 6.5 |
| 9 | 3 | Upper | Rinsed | Ljubljana | -2.37 | -0.2 | -0.04 | -0.20 | 0.00 | 7.3 |
| 10 | 1 | Inner | Unrinsed | Ljubljana | -2.12 | -0.14 | 0.00 | -0.15 | -0.03 | 13 |
| 11 | 2 | Inner | Unrinsed | Ljubljana | -1.96 | -0.09 | 0.04 | -0.13 | -0.05 | 11 |
| 12 | 3 | Inner | Unrinsed | Ljubljana | -2.01 | -0.13 | 0.04 | -0.14 | -0.06 | 12 |
| 13 | 1 | Outer | Unrinsed | Ljubljana | -2.22 | -0.09 | 0.02 | -0.10 | 0.04 | 8.2 |
| 14 | 2 | Outer | Unrinsed | Ljubljana | -2.31 | -0.12 | 0.04 | -0.17 | 0.04 | 9.3 |
| 15 | 3 | Outer | Unrinsed | Ljubljana | -2.25 | -0.14 | -0.02 | -0.15 | -0.02 | 7.1 |
| 16 | 1 | Upper | Unrinsed | Ljubljana | -2.17 | -0.1 | 0.01 | -0.09 | 0.03 | 9.1 |
| 17 | 2 | Upper | Unrinsed | Ljubljana | -2.08 | -0.15 | 0.00 | -0.17 | 0.01 | 8.6 |
| 18 | 3 | Upper | Unrinsed | Ljubljana | -2.32 | -0.18 | -0.02 | -0.17 | -0.03 | 7.5 |
| 19 | 1 | Inner | Rinsed | Idrija | -3.9 | -0.02 | 0.01 | -0.02 | -0.07 | 250 |
| 20 | 2 | Inner | Rinsed | Idrija | -3.23 | -0.02 | 0.00 | -0.06 | -0.03 | 250 |
| 21 | 3 | Inner | Rinsed | Idrija | -3.28 | 0.00 | 0.01 | -0.02 | -0.05 | 340 |
| 22 | 1 | Outer | Rinsed | Idrija | -3.6 | 0.00 | 0.02 | -0.02 | 0.00 | 280 |
| 23 | 2 | Outer | Rinsed | Idrija | -2.82 | -0.06 | 0.00 | -0.08 | 0.05 | 240 |
| 24 | 3 | Outer | Rinsed | Idrija | -3.21 | -0.05 | 0.03 | -0.03 | 0.05 | 170 |
| 25 | 1 | Upper | Rinsed | Idrija | -3.57 | -0.07 | -0.01 | -0.07 | -0.03 | 130 |
| 26 | 2 | Upper | Rinsed | Idrija | -4.07 | -0.07 | 0.03 | -0.12 | 0.04 | 84 |
| 27 | 3 | Upper | Rinsed | Idrija | -0.63 | -0.04 | 0.01 | -0.04 | 0.04 | 430 |
| 28 | 1 | Inner | Unrinsed | Idrija | -2.13 | -0.04 | 0.01 | -0.03 | 0.03 | 390 |
| 30 | 2 | Inner | Unrinsed | Idrija | -3.44 | -0.05 | 0.00 | -0.03 | 0.02 | 330 |
| 29 | 3 | Inner | Unrinsed | Idrija | -2.94 | -0.02 | -0.02 | -0.01 | -0.02 | 410 |
| 32 | 1 | Outer | Unrinsed | Idrija | -3.14 | -0.03 | 0.01 | -0.05 | 0.04 | 310 |
| 31 | 2 | Outer | Unrinsed | Idrija | -1.68 | -0.01 | 0.00 | -0.06 | -0.06 | 500 |
| 33 | 3 | Outer | Unrinsed | Idrija | -0.8 | -0.05 | 0.03 | -0.01 | -0.05 | 510 |
| 34 | 1 | Upper | Unrinsed | Idrija | -3.23 | -0.08 | 0.00 | -0.09 | -0.01 | 120 |
| 35 | 2 | Upper | Unrinsed | Idrija | 0.37 | -0.03 | 0.00 | -0.03 | -0.04 | 700 |
| 36 | 3 | Upper | Unrinsed | Idrija | 0.38 | -0.04 | 0.03 | 0.01 | 0.07 | 820 |


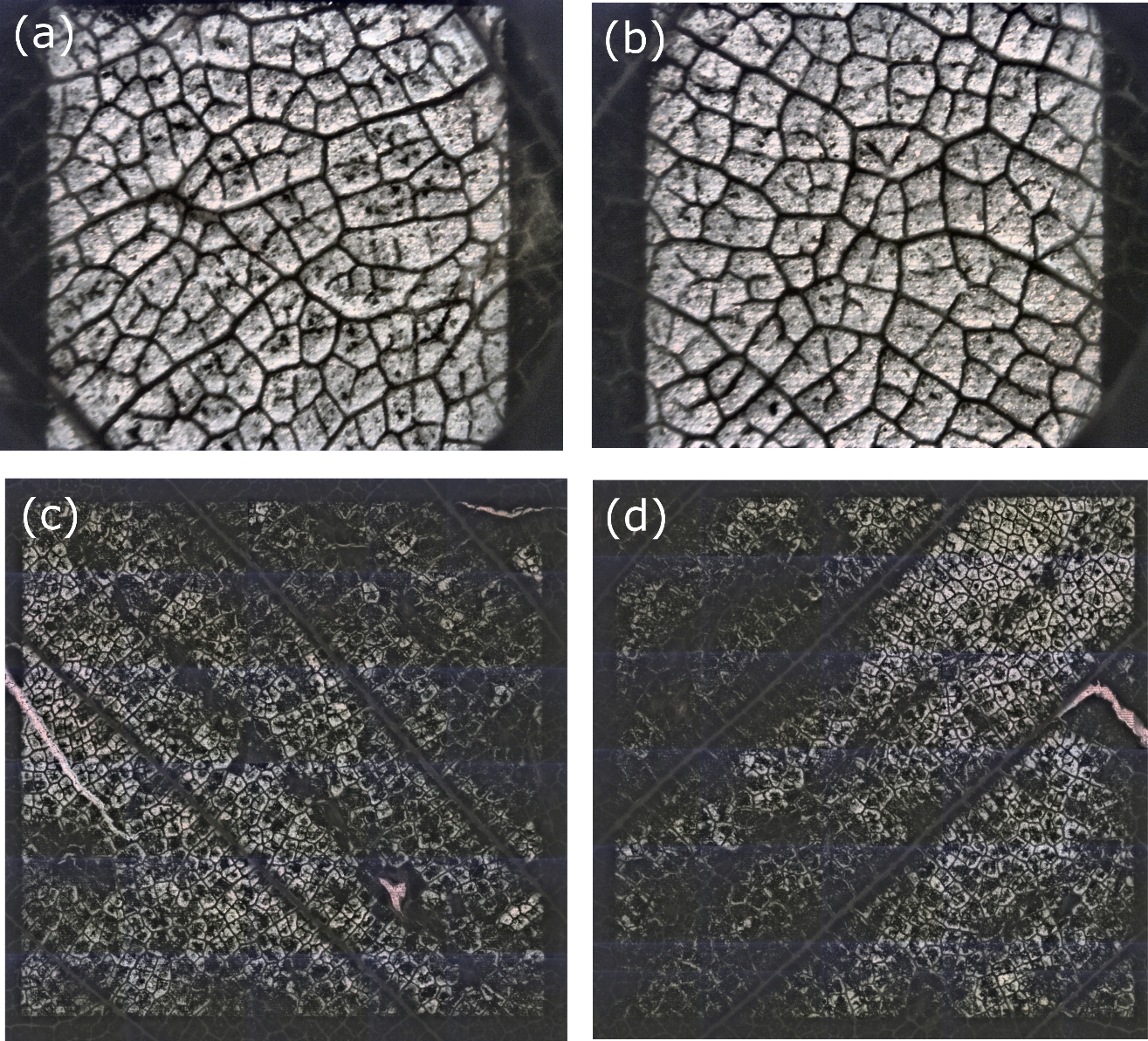


Figure S2. Photographic images of the ablated areas. Panels a and b depict ablated areas of 3 mm × 3 mm, while panels c and d show ablated areas of 10 mm × 10 mm. These images correspond to the regions analyzed using LA-ICP-MS for spatial distribution of Hg counts per pixel.

**Effect of sample pretreatment methods on residual moisture content in foliage samples.**

Ljubljana

Effect of sample drying on foliar residual moisture content

The sample drying method significantly influenced foliar residual moisture content in Ljubljana. For rinsed samples, room-drying retained an average moisture content of 6.6% ± 0.34% (1SD, n=3), which was notably higher than oven-dried samples (5.4% ± 0.40%, 1SD, n=3) and freeze-dried samples (5.0% ± 0.31%, 1SD, n=3). Statistical analysis revealed significant differences between room drying and oven drying (p=0.05) and between room drying and freeze drying (p<0.01), while the difference between oven drying and freeze drying was not statistically significant (p=0.20). Similarly, for unrinsed samples, room-drying showed an average moisture content of 5.7% ± 0.46% (1SD, n=3), compared to oven-dried samples (4.8% ± 0.06%, 1SD, n=3) and freeze-dried samples (4.8% ± 0.27%, 1SD, n=3). The differences were statistically significant between room drying and both oven drying (p=0.04) and freeze drying (p=0.01), but not between oven drying and freeze drying (p=0.41). Room drying therefore, retained approximately 19–37% more moisture compared to controlled methods of oven or freeze drying, likely due to lower evaporation efficiency under ambient conditions.

Effect of sample rinsing on foliar residual moisture content

Rinsing process however, impacted residual moisture content, with varying effects depending on the drying method. In room-dried samples, rinsing increased moisture content from 5.7% ± 0.46% (1SD, n=3) to 6.6% ± 0.34% (1SD, n=3), a 15.1% increase, which was statistically significant (p=0.03). Similarly, for oven-dried samples, rinsing elevated moisture content from 4.8% ± 0.06% (1SD, n=3) to 5.4% ± 0.40% (1SD, n=3), a 13% increase (p=0.04). For freeze-dried samples, the increase from 4.8% ± 0.27% (1SD, n=3) to 5.0% ± 0.31% (1SD, n=3) was minimal (3.8%) but still statistically significant (p=0.01). Rinsing introduced additional residual water, particularly in methods with limited evaporation efficiency, such as room drying or moderate-temperature oven drying. Freeze drying, being more efficient at removing water, was less affected by rinsing-induced moisture changes.

Idrija

Effect of sample drying on foliar residual moisture content

In the Idrija samples, drying methods significantly influenced residual moisture content. For rinsed specimens, room-dried samples had an average moisture content of 5.5% ± 0.24% (1SD, n=3), which was significantly higher than oven-dried samples (4.4% ± 0.40%, 1SD, n=3) and freeze-dried samples (4.7% ± 0.18%, 1SD, n=3). Statistical analysis showed significant differences between room drying and both oven drying (p=0.01) and freeze drying (p=0.03), while the difference between oven drying and freeze drying was not statistically significant (p=0.17). For unrinsed specimens, room-dried samples exhibited an average moisture content of 5.4% ± 0.19% (1SD, n=3), compared to oven-dried samples (4.4% ± 0.49%, 1SD, n=3) and freeze-dried samples (4.8% ± 0.48%, 1SD, n=3). Similar to rinsed samples, the differences were statistically significant between room drying and both oven drying (p=0.03) and freeze drying (p=0.03), but not between oven drying and freeze drying (p=0.19). These results indicate that room-dried samples retain approximately 15–25% more moisture than those dried using controlled methods, likely due to reduced evaporation efficiency under ambient conditions.

Effect of sample rinsing on foliar residual moisture content

On the other hand, rinsing had a minimal effect on residual moisture content in Idrija samples across all drying methods. For room-dried samples, rinsing increased moisture content slightly from 5.4% ± 0.19% (1SD, n=3) to 5.*5*% ± 0.24% (1SD, n=3), a negligible difference that was not statistically significant (p=0.44). Similarly, for oven-dried samples, rinsing resulted in an increase from 4.*4*% ± 0.49% (1SD, n=3) to 4.4% ± 0.40% (1SD, n=3), which was also not statistically significant (p=0.21). For freeze-dried samples, rinsing led to a minor decrease from 4.*8*% ± 0.48% (1SD, n=3) to 4.7% ± 0.18% (1SD, n=3), again showing no statistical significance (p=0.40). These findings suggest that rinsing does not introduce substantial additional water in Idrija foliage samples, regardless of the drying method used. This contrasts with other sites where rinsing has been shown to increase residual moisture content significantly, highlighting site-specific variations in sample behaviour.
